# PEGylated nano-graphene oxide as a nanocarrier for delivering mixed anticancer drugs to improve anticancer activity

**DOI:** 10.1038/s41598-020-59624-w

**Published:** 2020-02-17

**Authors:** Xibo Pei, Zhou Zhu, Zhoujie Gan, Junyu Chen, Xin Zhang, Xinting Cheng, Qianbing Wan, Jian Wang

**Affiliations:** State Key Laboratory of Oral Diseases, National Clinical Research Center for Oral Diseases, Department of Prosthodontics, West China Hospital of Stomatology, Sichuan University, Chengdu, Sichuan 610041 China

**Keywords:** Chemical modification, Cancer therapy

## Abstract

Due to their high specific surface area, graphene oxide and graphene oxide-base nanoparticles have great potential both in dual-drug delivery and combination chemotherapy. Herein, we developed cisplatin (Pt) and doxorubicin (DOX) dual-drug-loaded PEGylated nano-graphene oxide (pGO) to facilitate combined chemotherapy in one system. In this study, nano-sized pGO-Pt/DOX ranged around 161.50 nm was fabricated and characterized using zeta-potential, AFM, TEM, Raman, UV-Vis, and FTIR analyses. The drug delivery efficacy of Pt was enhanced through the introduction of pGO, and the final weight ratio of DOX: Pt: pGO was optimized to 0.376: 0.376: 1. *In vitro* studies revealed that pGO-Pt/DOX nanoparticles could be effectively delivered into tumor cells, in which they induced prominent cell apoptosis and necrosis and exhibited higher growth inhibition than the single drug delivery system or free drugs. The pGO-Pt/DOX induced the most prominent cancer cell apoptosis and necrosis rate with 18.6%, which was observed almost 2 times higher than that of pGO-Pt or pGO-DOX groups. in the apoptosis and necrotic quadrants *In vivo* data confirmed that the pGO-Pt/DOX dual-drug delivery system attenuated the toxicity of Pt and DOX to normal organs compared to free drugs. The tumor inhibition data, histopathology observations, and immunohistochemical staining confirmed that the dual-drug delivery system presented a better anticancer effect than free drugs. These results clearly indicated that the pGO-Pt/DOX dual-drug delivery system provided the means for combination drug delivery in cancer treatment.

## Introduction

To improve the efficacy and reduce side effects of anticancer drugs, nanoparticle drug delivery systems (DDSs) have been widely explored in the past few decades^1^. Graphene, one of the most popular nanoparticles, has attracted tremendous attentions in the DDSs field due to its unique physical and chemical properties^2^. Graphene is a two-dimensional single layer constituted of sp^2^-hybridized carbon, which could supply a excellent drug-load ability with its high specific surface area. As a derivatives of graphene, graphene oxide (GO), which could be detected to have hydroxyl, carbonyl, carboxyl and epoxide functional groups on thesurfaces of each shee^3^. The presence of those reactive functional groups imparts GO with excellent aqueous solubility, biocompatibility and multi-functionalities, which is essential for the delivery of anticancer drugs^4^.

Because of the unique properties of graphene oxide (GO), many efforts have been made to load anticancer drugs onto GO efficiently by either non-covalent or covalent bonds^5^. For instance, Dai and colleagues^6^ first attached hydrophobic aromatic molecules such as a camptothecin analogue (SN38) to the GO surface. The resulting GO-SN38 complex exhibits enhanced solubility and higher anticancer ability than the original SN38 prodrug does. In our previous study^7^, cisplatin (Pt) was carried by PEG-functionalized GO (pGO), and the as-prepared GO-Pt exhibits improved drug loading efficiency and enhanced toxicity to cancer cells *in vitro*. Cisplatin (Pt) is one of the most effective chemotherapeutic agents and has been used for treating different tumors, such as lung, neck and urinary bladder cancers^8–10^. The widely accepted mechanism is that cisplatin disrupts cell repair by cross-linking with DNA strands and ultimately causes cell death^11^. However, the potential disadvantages of using platinum-based drugs are the toxicities and acquired resistance^12^. Moreover, a single agent anticancer therapy may lead to tumor hypoxia that adversely impairs the therapeutic effects. Therefore, the combination of cisplatin with other anticancer drugs at an optimal dosage with rational design may be used^11^.

Although a large number of papers have used PEGylated GO as a carrier for anti-cancer treatment, most of these studies have used just one drug (such as DOX) as the active ingredient. In order to ensure the therapeutic effect, the dose of the single anticancer drug often requires a larger amount, which leads to more significant *in vivo* toxicity[]. Moreover, tumors have different tolerances to drugs, thus one single drug may not meet the goal of killing tumor cells. Therefore, a more advanced strategy to build carrier system is needed, which could integrate the safe dose and anti-cancer effect at the same time. Combination chemotherapy provides new ideas for anti-cancer treatment. In recent years, the combination chemotherapy has been adopted to build a standard clinical strategy against many types of cancer^13,14^. Because it is evidenced that tumorigenesis is a comprehensive result, and the therapeutic effects from single anticancer drug might be limited by this compensatory mechanism^15^. Hence, it is widely accepted that the combinatorial treatment of cancer with two or more drugs to inhibit different pathways improves drug efficacy, achieves synergistic effects and prevents drug resistance^16^. Doxorubicin (DOX) is a broad-spectrum antitumor chemotherapeutic drug commonly used in the treatment of a variety of cancers^17^. DOX has been combined with Pt for endometrial cancer therapy in phase III clinical trials, showing a significant therapeutic effect with increased cell toxicity^18^. Some of the nanoparticles have been developed for the loading of doxorubicin and cisplatin, for example, Yunfeng^15^ and Xueqiong^11^ prepared dual-drug (doxorubicin and platinum-base compounds) delivery system using hollow mesoporous silica and carboxymethyl chitosan nanoparticles, respectively. The results of both studies showed enhanced *in vitro* anticancer properties without *in vivo* study results, and the drug loading efficacy (DLE) of the dual drugs is relatively low. Therefore, there are still many challenges such as low DLE, and unclear *in vivo* behaviors should be overcome. To decrease the toxicity of DOX and Pt combination, a nanoscale drug delivery system (like GO) should be employed.

As mentioned above, the ultra-high surface area and abundant functional groups of GO can provide a possible approach by incorporating two drugs into one nanocarrier. However, few reports were found in which fabricated a controlled loading of Pt and DOX drugs using pGO nanoparticles. Therefore, we hypothesized that it would be beneficial to develop a GO-based drug delivery system for Pt and DOX combination chemotherapy. In this study, the polymeric Pt prodrug was first prepared and covalently attached to PEGylated GO nanosheets (pGO) via amino-bonding reaction to form pGO-Pt nanoparticles. Then, DOX was loaded onto pGO-Pt to form the dual-drug delivery system (pGO-Pt/DOX) through a non-covalent reaction via π-π interactions. Finally, the pGO-Pt/DOX was completely characterized. The advantage of this dual-drug delivery system was presented by detecting its drug delivery efficacy, drug releasing and cytotoxicity against cancer cells *in vitro* and *in vivo*.

## Results

### Synthesis, preparation and characterization

**Figure 1:**
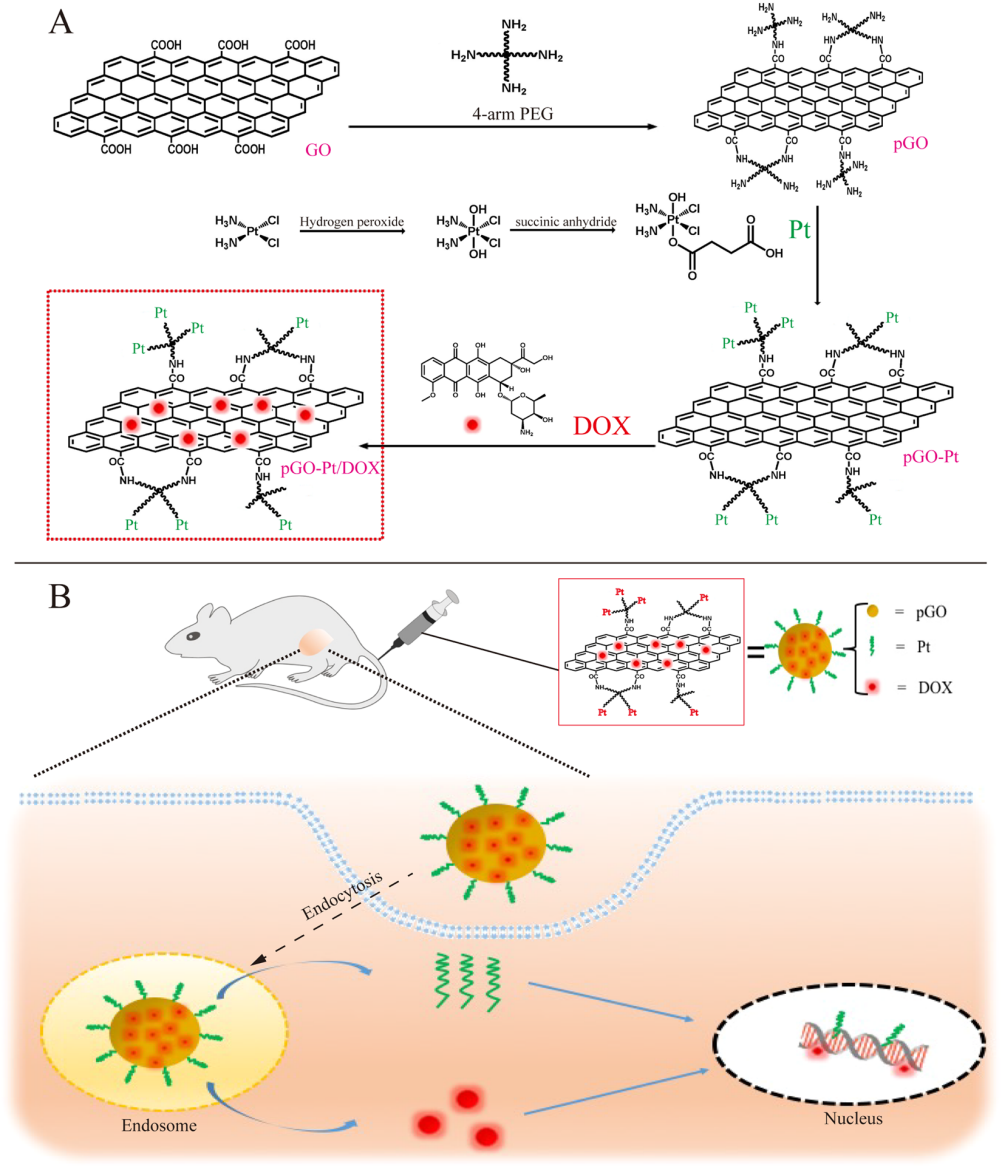
Schematic illustration of co-delivery of Pt and DOX by PEGylated nano-graphene oxide for improving anticancer activity. (**A**) The synthesis of pGO-Pt/DOX dual-drug delivery system, which included the step of covalent functionalization by PEG forming pGO nanoparticle, followed by covalent functionalization by Pt and non-covalent functionalization by DOX. (**B**) Schematic illustration of tumor-targeted pGO-Pt/DOX nanoparticle to achieve superior anticancer efficacy by generating DNA lesions (Pt) and preventing the DNA replication (DOX).

Synthesis of dual-drug delivery system (pGO-Pt/DOX) consisted of the following steps (Fig. 1A). Firstly, PEGylated nano-graphene oxide (pGO) was synthesized with PEG covalent binding to GO via amide linkage^12^. As shown in Fig. S1 and Table 1, nano-sized (146.1 nm) pGO was fabricated, and the zeta potential of GO increased from −36.8 ± 7.3 mV to −16.8 ± 0.87 mV, indicating that the 4-arm PEG-amine banded and neutralized some negatively charged carboxylic acid groups in GO^12^.

**Table 1:** Zeta Sizer analysis showed the size and zeta-potential analysis showed the zeta potential value of GO, pGO, pGO-Pt, and pGO-Pt/DOX.

	GO	pGO	pGO-Pt	pGO-Pt/DOX
Mean size (nm)	140.85	146.10	154.39	161.50
Zeta-potential (mV)	−36.8 ± 7.3	−16.8 ± 0.87	−19.5 ± 1.2	−17.9 ± 1.5

(Data presented as mean ± SD, n = 3).

**Figure 2:**
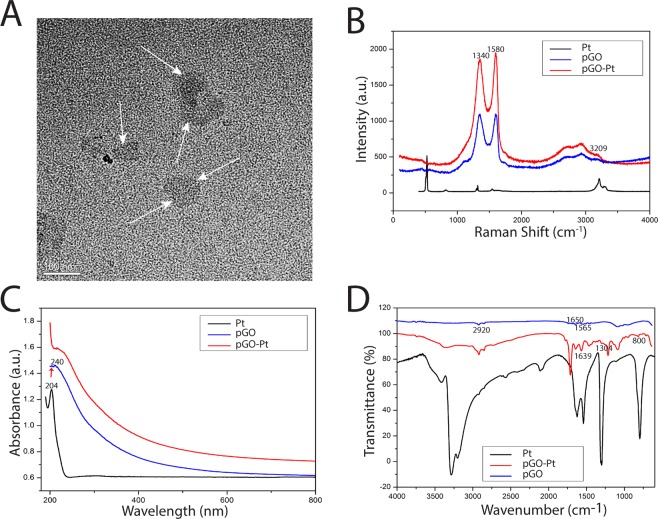
Characterization of pGO-Pt, Pt and pGO. (**A**) TEM image of pGO-Pt. (**B**) Raman spectrum of pGO-Pt, Pt and pGO. (**C**) UV-Vis spectra of pGO-Pt, Pt and pGO. (**D**) FTIR spectra of pGO-Pt, Pt and pGO. White arrows show the existence of Pt attached onto pGO.

Then, cisplatin was reacted with H_2_O_2_ and succinic anhydride to form Pt(NH_3_)_2_Cl_2_(OOCCH_2_CH_2_C-OOH)(OH) (abbreviated as Pt(IV) or Pt), and the Pt-loaded pGO (pGO-Pt) nanoparticles was accomplished by a covalent reaction^19^. The coupling reaction was achieved in the presence of EDC and NHS as the activating agent for the carboxylic group at room temperature (Fig. 1A). As shown in Fig. 2 and Table 1, nanoscale pGO-Pt was achieved (Fig. 2A) with the size slightly increased from 146.10 nm (pGO) to 154.39 nm after the loading of Pt (Table 1). Furthermore, the attachment of Pt, which could be prompted in TEM images (Fig. 2A), because of a portion of positively charged amino groups on pGO, the Zeta potential showed a decreased trend (Table 1)^20^. Raman spectroscopy (Fig. 2B) gives a nondestructive way to characterize the graphene and its derivatives. In Fig. 2B, the G and D bonds of graphene could be found at 1580 and 1340 cm^−1^, indicating that no substantial structural damage emerged during the modification procedure. A trend was observed from pGO to pGO-Pt, with a tiny difference at 3209 cm^−1^, which was in good agreement with the peak of Pt. The results indicated that Pt was attached to the pGO without affecting the basic structure. In Fig. 2C, the attachment of Pt onto pGO was evident from the UV-Vis spectra of pGO-Pt solution, which showed the characteristic absorption peaks of Pt at 204 nm. FTIR spectra (Fig. 2D) of pGO, pGO-Pt, and Pt nanoconjugates were also collected. The characteristic CO–NH at 1565 and 1650 cm^−1^ and NH at 2920 cm^−1^ were detected in pGO, which may owing to the formation of amide linkage between GO and polyethylene glycol^21,22^. The spectrum of pGO-Pt was almost the same as that of pGO, however, the typical but weakened Pt absorption (1639, 1304, and 800 cm^−1^) was observed^23,24^. All these results indicated the successful incorporation of Pt into pGO.

**Figure 3:**
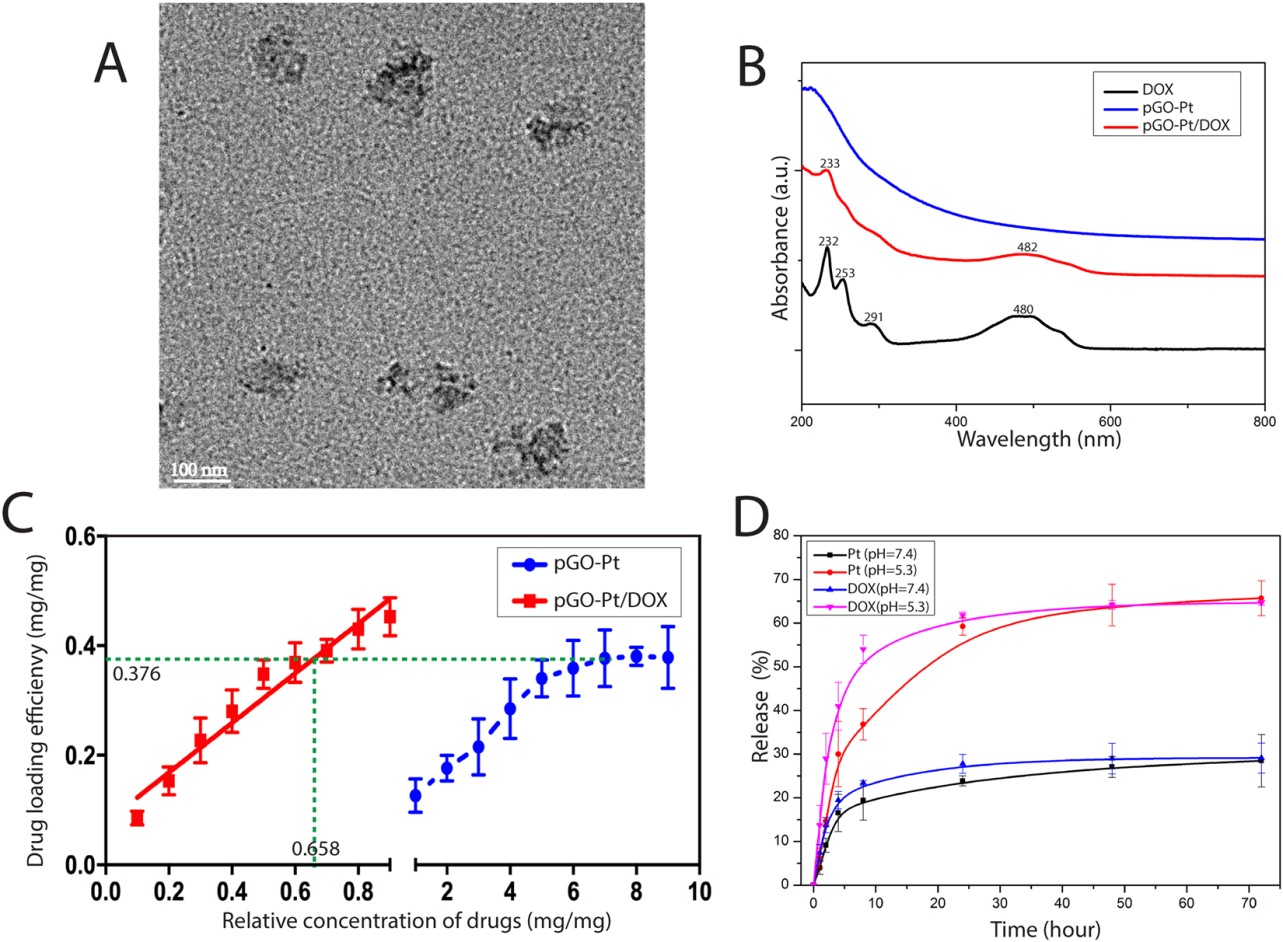
Characterization of pGO-Pt/DOX nanoparticles. (**A**) TEM image of pGO-Pt/DOX. (**B**)UV-Vis spectra of pGO-Pt/DOX, pGO-Pt, Pt and DOX. (**C**) The drug loading efficiency (DLE) of pGO-Pt/DOX, the saturated DLE of Pt was at around 0.376 mg/mg. we managed to optimize the finally weight ratio of DOX: PT: pGO = 0.376: 0.376: 1. Thereby, the DLE of DOX reached to 0.376 mg/mg when the initial concentration was 0.658 mg/mL (the including-pGO concentration of 1.0 mg/mL). (**D**) The release profile of Pt and DOX from pGO-Pt/DOX in buffers at pH 5.3 and 7.4.

Finally, in Fig. 1, the doxorubicin hydrochloride (DOX) was adhered to pGO-Pt nanoparticles by a π-π stacking interaction between the large π conjugated from GO and quinone portion of DOX^25^. As shown in Fig. 3A, the dispersive pGO-Pt/DOX nanoparticles with a narrow distribution of particle sizes were achieved. The size of pGO-Pt/DOX (159.2 ± 7.0 nm) was slightly increased compared to that of pGO-Pt (151.1 nm, Table 1). Furthermore, the slightly increased zeta potential might be attributed to the addition of amino groups in DOX after the conjugation of DOX to pGO-Pt nanoparticles^26^. These could be evidence for the successful conjugation. Then, more results of the interaction between pGO-Pt and DOX was provided by UV-Vis absorption spectra (Fig. 3B,C). In Fig. 3B, the absorption peaks of DOX were located at 232, 253, 291 and 480 nm^27^. After forming the dual-drug system, the UV–Vis spectra of pGO-Pt/DOX not only confirmed the stacking of DOX onto pGO-Pt nanoparticles but also showed the red-shift due to the interaction^28^. All these results demonstrated that DOX molecules were successfully loaded onto pGO-Pt, and the nanoscale dual-drug delivery system was formed.

### Characterization of dual-drug loading

With the large specific area, graphene oxide is considered to possess excellent loading behavior^29^. The DLE of the nanoparticles was calculated with different initial cisplatin and DOX concentrations relative to the same pGO concentration (including -pGO concentration of 1.0 mg/mL). As shown in Fig. 3C, the initial Pt/pGO reaction mass ratio of 1:1 to 7:1 resulted in a final Pt drug loading efficiency increasing almost from 0.126 to 0.376 mg/mg. However, no considerable DLE increase occurred when the Pt/pGO ratio was higher than 7:1, indicating that the saturated DLE of Pt was at approximately 0.376 mg/mg. These results showed that the GO-based nanoparticles could load Pt with exceptionally high DLE compared with many other common nanoparticles such as carbon nanotubes (0.21 mg/mg)^30^ and gelatin hydrogels (0.1 to 0.3 mg/mg)^31^. Then, DOX was loaded onto pGO-Pt nanoparticles to form the dual-drug delivery system (pGO-Pt/DOX), and we managed to optimize the final weight ratio of DOX: Pt: pGO to 0.376: 0.376: 1. In Fig. 3C, the DLE of DOX increased linearly with the increasing of initial DOX concentration, and the linear relationship could be described by the following typical equation: Y = 0.078 + 0.453X (R^2^ = 0.955). Thereby, according to the formula, the DLE reached to 0.376 mg/mg when the initial concentration was 0.658 mg/mL (the including-pGO concentration of 1.0 mg/mL).

### pH related dual-drug release

Drug release experiments for the synthesized dual-drug delivery system pGO-Pt/DOX were performed at 37 °C, pH of 7.4 and 5.3^32^. ICP-OES and UV–Vis spectroscopy were used to determine the amount of Pt and DOX released, respectively. Figure 3D showed that Pt and DOX released at a controlled and slow manner from pGO-Pt/DOX system, to 28.4% and 29.1%, respectively, after 72 h. On the other hand, the drug release rate was higher at pH 5.3. The results indicated that the amount of released Pt and DOX reached 30.0% and 41.0% from pGO-Pt/DOX system in first 4 h at pH 5.3, while it took more than 72 h at pH 7.4. Moreover, approximately 65.7% (Pt) and 64.6% (DOX) of the total bound drugs were released from dual-drug delivery system after 72 h at pH 5.3. Similar results were also observed by Haihua Xiao (for Pt release)^33^ and Jianmin Shen (For DOX release)^34^. These results suggested that both Pt and DOX release kinetics from pGO-Pt/DOX nanoparticles were pH-dependent, and such environmentally sensitive behavior could prevent drug loss during delivery and enhance drug release after reaching the tumor site.

### *In vitro* cell viability and cytotoxicity

**Figure 4:**
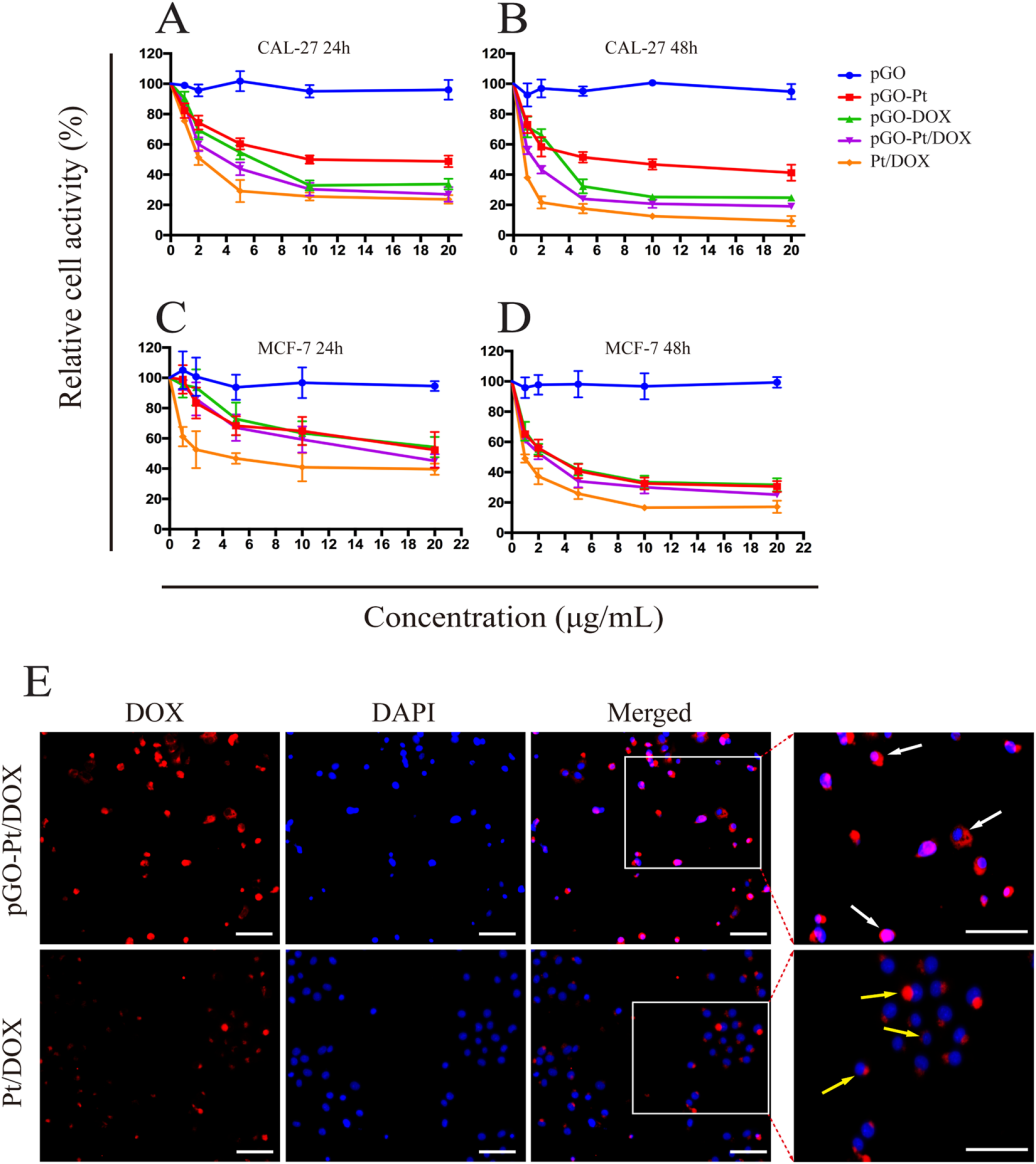
Cytotoxicity assay of CAL-27 and MCF-7 cancer cells with different concentrations (1–20 μg/mL) of PBS, pGO, pGO-Pt, pGO-DOX, pGO-Pt/DOX, and Pt/DOX mixture. The cell viability of PBS was set as 100%. Relative cell viability of CAL-27 cells for (**A**) 24 h and (**B**) 48 h after treatment with pGO, pGO-Pt, pGO-DOX, pGO-Pt/DOX, and Pt/DOX mixture. Relative cell viability of MCF-7 cells for (**C**) 24 h and (**D**) 48 h after treatment with pGO, pGO-Pt, pGO-DOX, pGO-Pt/DOX, and Pt/DOX mixture (n = 3). (**E**) Cellular uptake of DOX-labeled pGO-Pt/DOX and Pt/DOX mixture by CAL-27 human squamous cell carcinoma cell line.

To investigate *in vitro* the anticancer effect of pGO-Pt/DOX nanoparticles, the cell viability and cytotoxicity of PBS, pGO, pGO-Pt, pGO-DOX, pGO-Pt/DOX, and Pt/DOX mixture (1–20 μg/mL) were tested in CAL-27 and MCF-7 cancer cells. The cell viability of PBS was set as 100%. In Fig. 4A–D, the empty pGO revealed a high viability (above 90%) to CAL-27 and MCF-7 cells at 1–20 μg/mL after 24 and 48 h, and even at a concentration as high as 100 μg/mL (Fig. S2), the L929 cell viability still reached 94.3%. The results indicated that the PEG-functionalized GO is a safe drug carrier^2,4,7,26^. Then, the cell viability of all drug delivery systems and pure drug samples exhibited a dose-dependent pattern after incubation for 24 and 48 h. It is encouraging that the anti-tumor activity of the dual-drug delivery system (pGO-Pt/DOX nanoparticles) was obviously higher than that of the single-drug delivery system (pGO-Pt and pGO-DOX), indicating that the anti-tumor ability might be enhanced by using the confederate delivery of two drugs. The IC_50_ values of the drug delivery system and mixed drug were assessed in Table 2 and the following results were observed: First, the IC_50_ value of pGO-Pt/DOX was higher than that of pGO-Pt and pGO-DOX at 24 h and 48 h for both CAL-27 and MCF-7 cells; Second, the IC_50_ value of pGO-Pt/DOX was lower than that of mixed drug at 24 h for both CAL-27 and MCF-7 cells. However, pGO-Pt/DOX displayed a comparable IC_50_ value to mixed drug for the CAL-27 cells after 2 days. The reason is that the dual-drug delivery system was first disassembled after being internalized by cancer cells (Fig. 1B). Once enough drug released and accumulated within the tumor cells, the anticancer activity would be improved^35^. Moreover, the amount of the drugs in mixture drug (Pt/DOX) is 2.33-fold higher than the drugs in pGO-Pt/DOX (weight ratio of DOX: Pt: pGO = 0.376: 0.376: 1), that means 2.33 times the amount of mixture drug could only obtain comparable cytotoxicity compared with pGO-Pt/DOX system. In view of the above results, we used a more sensitive cancer cell line (CAL-27) for further studies.

**Table 2:** IC 50 of all groups for Cal-27 and MCF-7 cells (μg/mL).

Groups	24 h		48 h	
	Cal-27	MCF-7	Cal-27	MCF-7
pGO-Pt	15.07	22.03	10.77	3.03
pGO-DOX	9.99	22.48	4.89	3.22
pGO-Pt/DOX	7.28	14.49	1.24	2.14
Pt/DOX	3.31	3.58	0.74	0.80

### *In vitro* cellular uptake

Cellular uptake of DOX-labeled pGO-Pt/DOX and Pt/DOX mixture by CAL-27 human squamous cell carcinoma cell line was examined by fluorescence imaging (Fig. 4E). When CAL-27 cells were incubated with pGO-Pt/DOX, highlighted red fluorescence (DOX) was observed in the nucleus and cytoplasm. However, when CAL-27 cells were cultured with the Pt/DOX drug mixture, the red fluorescence mainly appeared in the cytoplasm, with obviously reducing of fluorescence intensity in the cytoplasm and nucleus. The results illustrated that the GO-based dual-drug delivery system could be effectively delivered into targeted tumor cells due to their higher efficiency of endocytosis than small molecular drugs^36^.

### *In vitro* cell apoptosis assay

**Figure 5:**
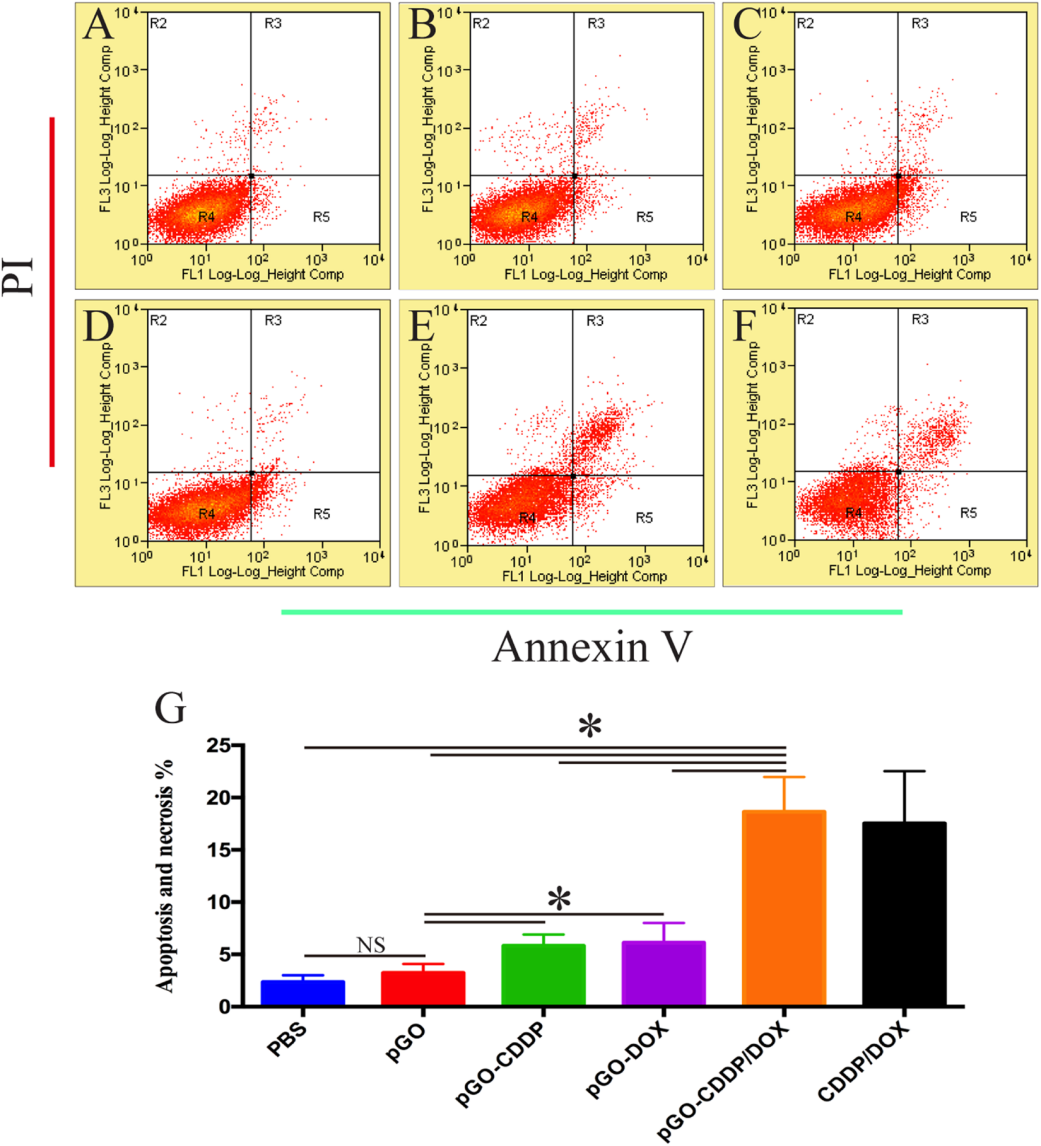
Cells were incubated with (**A**) PBS, (**B**) pGO, (**C**) pGO-Pt, (**D**) pGO-DOX, (**E**) pGO-Pt/DOX, and (**F**) Pt/DOX mixture. Four hours later, cells were double-stained with Annexin V/PI and analyzed by FACS. (**G**) The quantitative analysis of cell apoptosis. Bars, mean ± SD (n = 6). *p < 0.05; NS p > 0.05 compared with other group.

The result of further cell apoptosis evaluation are shown in Fig. 5. After incubating with the drug carrier for 4 h, the pGO showed a negligible effect on cell apoptosis and necrosis, revealing the excellent biocompatibility. And pGO-Pt and pGO-DOX nanoparticles induced 1.8 and 1.9 times higher cell apoptosis and necrosis than that of the pGO, respectively. Furthermore, pGO-Pt/DOX showed the highest cell apoptosis and necrosis compared with any other groups, which achieved 18.6% from data analysis (Fig. 5E)^37^. Although the mixed drug induced less cell apoptosis and necrosis, no significant differences were found between the pGO-Pt/DOX and Pt/DOX mixtures. According to the apoptosis assays, the pGO-Pt/DOX showed markedly apoptotic and necrotic effects, which verified the conclusion of CCK-8 assay (Fig. 4A,B) and fluorescence imaging (Fig. 4E).

### *In vivo* antitumor efficacy

Our *in vitro* studies suggest pGO-Pt/DOX nanoparticles might has better anti-cancer efficacy. The CAL-27 tumor models were generated by subcutaneous injection of CAL-27 cells into nude mice. After the tumor volumes reached 50–100 mm^3^, mice were subjected to intravenous administration with drugs every 3 days, for a total of seven injections.

**Figure 6:**
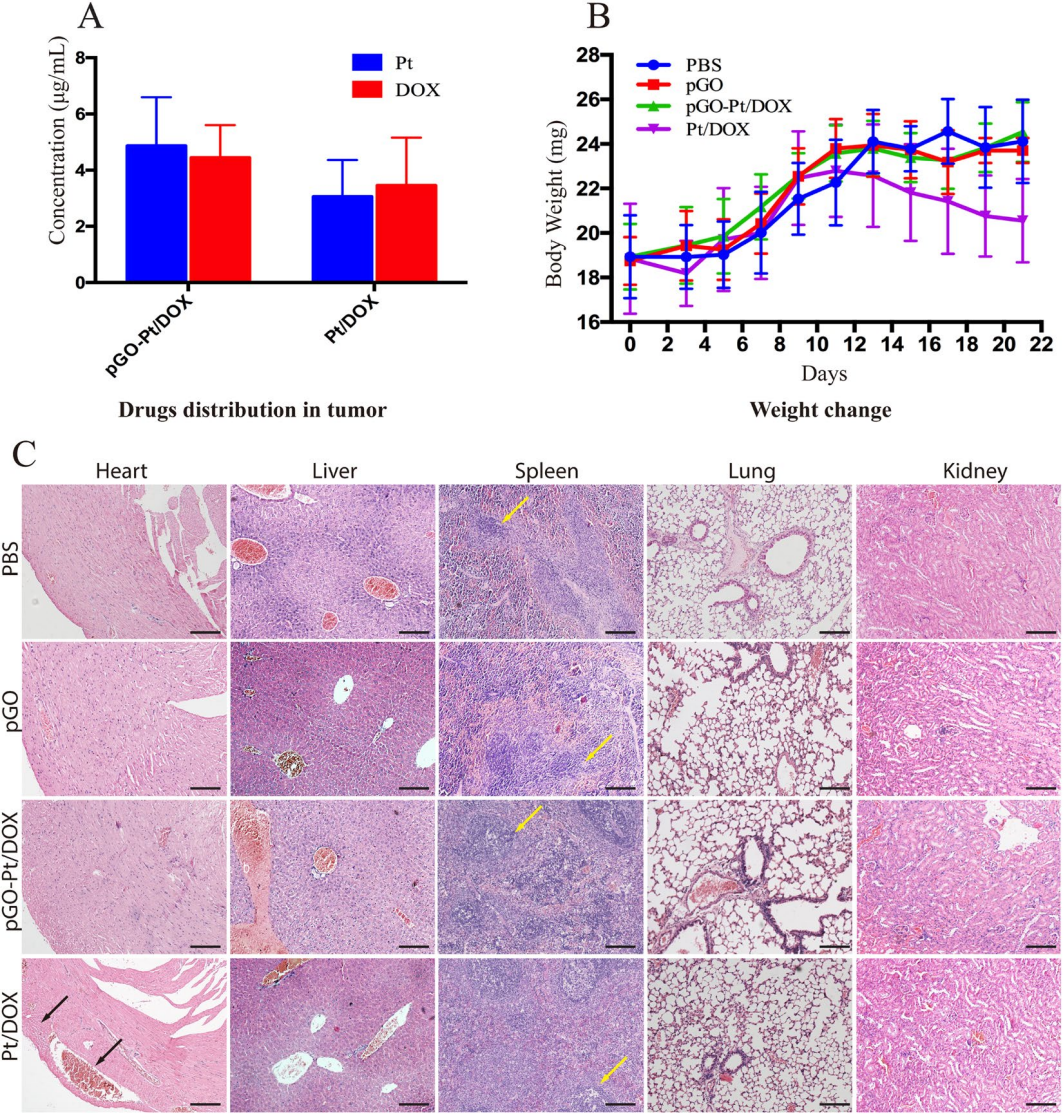
(**A**) Quantification analysis of both Pt and DOX amounts in tumor after 12 h post-injection of pGO-Pt/DOX and Pt/DOX mixture, respectively. Bars, mean ± SD (n = 6). (**B**) Body weight curves after treatment with PBS, pGO, pGO-Pt/DOX, and Pt/DOX mixture, respectively. Bars, mean ± SD (n = 6). (**C**) H&E staining images of major organs (heart, liver, spleen, lung and kidney) after different treatments. Black arrows represent myocardial congestion and cardiomyocyte hypertrophy. Yellow arrows represent splenic nodule. Scale bar: 100 μm.

The effective tumor targeting of pGO-Pt/DOX was performed by a quantification analysis of both Pt and DOX amounts in tumor after 12 h post-injection. As shown in Fig. 6A, the concentrations of Pt and DOX in tumor for Pt/DOX mixture administration group were 3.06 and 3.45 μg/mL, respectively, while the concentrations reached 4.87 and 4.43 μg/mL for Pt and DOX dual-drug delivery system, indicating that the amounts of Pt and DOX accumulated in the tumor for pGO-Pt/DOX were slightly higher than that for the Pt/DOX mixture. It should be noted that the amount of the drugs in the Pt/DOX mixture is 2.33-fold higher than that in pGO-Pt/DOX. Therefore, the above results suggested that dual-drug delivery system could accumulate in the tumor cells more efficiently.

The change in body weight was monitored to investigate the drug safety throughout the therapeutic period^20^, and the results were depicted in Fig. 6B. The body weights in the pGO-DOX/Pt and pGO groups showed a slow and sustained increase with no indication of toxicity versus the control group. However, mice receiving Pt/DOX mixture showed obvious weight loss, implying the side effects were produced by the mixture drugs. The typical H&E staining slices of organs were also monitored after the treatment (Fig. 6C). No significate histopathological changes were found in the main organs from the pGO-DOX/Pt, pGO and control groups (PBS). However, a cardiac section from Pt/DOX-treated sample showed marked damage changes including myocardial congestion and cardiomyocyte hypertrophy, indicating a considerable amount of DOX accumulated in heart tissue and caused evident cardiac toxicity^38^. Moreover, the obvious histopathological changes of the spleen were found in Pt/DOX-treated mice, suggesting the accumulation of Pt^20,39^. Additionally, blood chemistry was also introduced to analyze the hepatic function, nephrotoxicity, and cardiac damage of tumor-bearing nude mice that were treated with PBS, pGO, pGO-Pt/DOX and Pt/DOX. The data were collected in Table 3. Mice treated with pGO and pGO-Pt/DOX showed no significant differences in all these parameters compared with controls, indicating no obvious toxicity to the liver, kidney and heart, which was consistent with H&E staining results. As suggested by H&E staining, the cardiac damage of Pt/DOX-treated mice agreed with the significant changes of CK and LDH (Table 3). Although no obvious histopathological changes in kidney were observed, the BUN levels of Pt/DOX mixture group were 3-fold higher than that of control group (PBS), and this increase in BUN might indicate the beginning of nephrotoxicity and was consistent with previous study^40^. The above results indicated that the dual-drug delivery system substantially attenuated the toxicity of Pt and DOX to normal organs compared to free drugs.

**Table 3:** Biochemical assay in serum (Data presented as mean ± SD, n = 6).

Parameters	PBS	pGO	pGO-Pt/DOX	Pt/DOX
ALT (alanine aminotransferase) (U/L)	6.8 ± 6.6	38.6 ± 13.0	39.6 ± 5.9	45.6 ± 11.3
AST (aspartate aminotransferase) (U/L)	147 ± 28	143 ± 26	164.6 ± 23	147 ± 51
ALP (alkaline phosphatase) (U/L)	70.6 ± 9.5	72.2 ± 22.8	74.2 ± 17.3	74.2 ± 19.1
BUN (blood urea nitrogen) (mmol/L)	7.7 ± 1.9	8.8 ± 4.2	9.6 ± 3.8	24.4 ± 8.9*
CK (creatine kinase) (U/L)	452 ± 60	479 ± 70	439 ± 111	627 ± 106*
LDH (lactate dehydrogenase) (U/L)	491 ± 66	498 ± 85	478 ± 118	652 ± 165*

**Figure 7:**
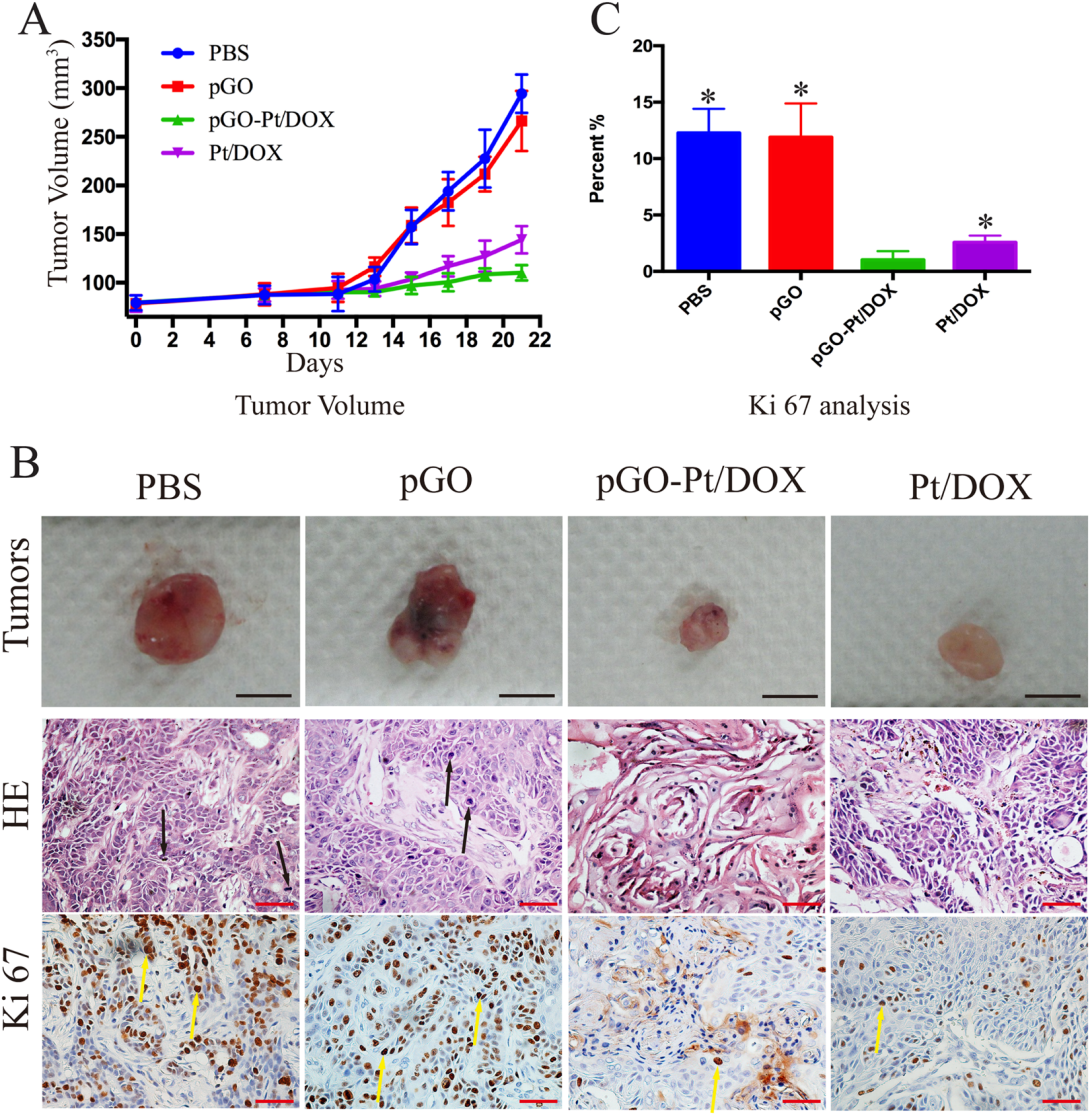
*In vivo* antitumor evaluation. (**A**) Tumor growth curves of different groups after treatment with PBS, pGO, pGO-Pt/DOX, and Pt/DOX mixture, respectively. Bars, mean ± SD (n = 6). **(B**) Representative photos, H&E staining and immunohistochemical staining Ki-67 of tumor tissues get from mice after various treatments. Black arrows represent the huge cell nuclei. Yellow arrows represent the Ki-67 active cells. Black Scale bar: 5 mm; Red Scale bar: 50 μm. (**C**) Quantitative analysis the percentage of Ki-67 active cells in tumors. Bars, mean ± SD (n = 6). *p < 0.05.

Then we further examined the xenograft tumor growth of CAL-27 cancer following intravenous administration of PBS, pGO, pGO-Pt/DOX and Pt/DOX mixture. The tumor volumes were recorded in Fig. 7A. After 3 weeks, there was no significant difference in tumor sizes between the pGO group and the control group (PBS). Mice treated with Pt/DOX mixture developed tumor with an average size of 144 mm^3^, indicating the inhibition rate reached to 51%. While the treatment with pGO-Pt/DOX led to more effective inhibition of tumor growth (63%). The results were consistent with the *in vitro* cell study and the pictures shown in Fig. 7B. The dark color of the tumor in pGO- and pGO-Pt/DOX-treated groups (Fig. 7B) might indicate the accumulation of GO. The hematoxylin and eosin (H&E)-stained sections of tumor tissue were also shown in Fig. 7B. The tumors of the control (PBS) and pGO groups exhibited non-uniform dense cellularity and huge cell nuclei, indicating the tumors from these two groups have vigorous proliferative capability^20^. Compared with the PBS-treated group, both therapeutic groups showed uniform dense cellularity and less huge cell nuclei, which indicated that treating tumor with Pt and DOX resulted in tumor cells inhibition^34^. It was noteworthy that fewer tumor cells, smaller cell nuclei, and the formation of tumor nests were observed for the mice treated with the dual-drug delivery system. Furthermore, to evaluate the effect of the dual-drug delivery system on tumor proliferation, immunohistochemical staining Ki-67 was performed. As shown in Fig. 7B,C, the percentage of Ki-67 active cells was significant lower in pGO-Pt/DOX group (1.03 ± 0.76%) than that in PBS (12.27 ± 2.15%), pGO (11.89 ± 3.01%), and Pt/DOX mixture (2.57 ± 0.61%) groups. These results indicated that pGO-Pt/DOX could suppress tumor cell proliferation.

## Discussion

Here, we successfully synthesized a dual-drug delivery system using PEG-functionalized graphene oxide (pGO) and anticancer drugs (Pt and DOX). We demonstrated that pGO nanosheets had enhanced biocompatibility and could be used as a safe nanocarrier. Moreover, the surface modified dual-drug system (pGO-Pt/DOX) showed high DLE, controlled release and high cellular uptake properties, and could enhance antitumor effect both *in vitro* and *in vivo*.

### PEG functioned GO is a safe drug delivery carrier

As graphene oxide (GO) has functional groups for better bonding, is excellently dispensability within water, and is easy to manufacture^3^, it is considered highly interesting in biomedical fields including drug delivery^4^. In this study, we introduced GO nanosheets as the carrier of anticancer drugs to fabricate a dual-drug delivery system (Fig. S1). However, nanomaterials might present side effects sometimes^41,42^, and the biological toxicity of GO remains controversial. For example, Matthew and colleagues^43^ directly administered the solution of GO into the lungs of mice could cause lung injury. On the other hand, Yanli and colleagues^44^ explored the toxicity of graphene oxide (GO) by analyzing the influences of GO on morphology, viability and membrane integrity, and reported that GO had no obvious cytotoxicity for A549 cells. Moreover, Liu and colleagues^45^ indicated that PEGylated GO showed distinctive *in vivo* behaviors, such as reduced reticuloendothelial system accumulation and improved tumor passive targeting effect.

Interestingly, the results in Fig. 4 showed that PEG-functionalized GO (pGO) revealed a high viability to both CAL-27 and MCF-7 cells at 1–20 μg/mL after 24 and 48 h. The viability of L929 cells was still as high as 94.3% when the concentration reached 100 μg/mL (Fig. S2). The pGO also showed a negligible effect on cell apoptosis and necrosis in cell apoptosis assay (Fig. 5). Furthermore, in mice treated with pGO, there was little difference in body weights (Fig. 6B), blood chemistry (Table 3) and hematoxylin-eosin (HE) staining for the main organ (Fig. 6C) compared to that of PBS group. These results collectively indicated that pGO was highly biocompatible and could be used as the nanocarrier of dual-drug delivery system, which is consistent with the pGO drug delivery studies^2,4,7,26,36,45^. The possible reasons of the highly biocompatibility of pGO were suggested as follows: (1) Surface modification could enhance biocompatibility. Unlike Matthew and colleagues, who used nonfunctional GO in their research, many studies have indicated that PEG modification could lower the cytotoxicity of GO. For instance, it has been shown that pGO was not toxic against Hela or MCF-7 cells^46^ and had no effect on cell viability even at concentrations up to 100 mg/L^6^, which is consistent with our results. Liu and colleagues^45^ also found that pGO (20 mg/kg) intravenously administered into mice did not cause any organ damage. (2) The nanoscale size of GO was crucial for biological response. In our study, the diameter of all the particles were approximately 140 nm (Table 1), which is considered nanoscale^47^. Su and colleagues^48^ showed that both nanoscale (350 nm) and microscale (2 μm) GO could be taken up by macrophages, but only microscale GO initiated a severe inflammatory responses. Based on the above discussion, we suggested that pGO could be used as a safe and effective nanocarrier for dual-drug delivery system.

### Dual-drug delivery system possesses high DLE, superior cell uptake and controlled release property

The discovery and application of pGO as a carrier for drug loading extending the chemotherapeutic filed^49^. Anticancer drugs, both hydrophobic (Pt) and hydrophilic (DOX), were effectively loaded and protected by the dual-drug delivery system in this study (Figs. 2 and 3). Previous studies showed that DOX has been loaded onto GO by physisorption or chemical conjugation with a high DLE^22^. However, for the delivery of cisplatin, although many drug delivery system^50–53^ have been investigated, the DLE was relatively low. Therefore, building a superior carrier for Pt with high DLE has been the primary challenge. Xueqiong^11^ prepared doxorubicin and platinum-base compounds delivery system using carboxymethyl chitosan nanoparticles. Their result showed anticancer effect with the drug loading efficacy (DLE) relatively low. In addition, the size of chitosan nanoparticles (274 nm) was bigger that the pGO (159 nm) in this study, making it less susceptible to achieve endocytosis which may reduce chitosan nanoparticles’ anticancer effect. On the other hand, the mesoporous silica nanoparticles (MSN) also had been used to build a doxorubicin and platinum-base compounds delivery system^13^. However, using pGO to transport DOX and Pt could achieve a loading rate of 0.367 mg/mg, which is significantly higher than that of MSN. Moreover, the pGO-Pt/DOX system showed effective antitumor ability *in vivo*, while the above tow studies only showed enhanced *in vitro* anticancer properties. In our study, Pt was first covalently bound with pGO followed by the non-covalent loading of DOX (Fig. 1). The results (Fig. 3C) showed that the pGO-based nanoparticles could load Pt with higher DLE (0.376 mg/mg), compared with carbon nanotubes (0.21 mg/mg)^30^ and gelatin hydrogels (0.1 to 0.3 mg/mg)^31^. The high DLE could be attributed to the large and negatively charged surface of pGO^4^ (Table 1).

A controlled drug release profile is essential to prolong the plasma half-life of the drug^54^. It is well known that the pH in tumor cell lysosomes, endosomes and tumor microenvironment is acidic^26,55^. Therefore, it is also critical for the drug delivery system to respond to PH. Our study (Fig. 3D) showed that the Pt and DOX release rates were significantly higher at pH 5.3 versus pH 7.4, which suggested that both Pt and DOX release kinetics from dual-drug delivery system were pH-dependent. This pH-dependent release behavior could enhance the permeability and retention (EPR) effect of solid tumor^56^. The EPR effect could be achieved by the following reasons: first, there is a leaking vasculature in the tumor, and the holes in the tumor vessel wall range from 200 nm to 2 μm with an average of about 400 nm^57^. Many studies suggest that 100–200 nm diameter nanoparticles are easier to supply blood to tumors^24,57^. Secondly, the negative charge (Table 1) and PEG segments (Fig. 1) of the drug delivery system prevented themselves from being recognized and prolonged their circulation time in blood, which in turn enhanced the EPR effect^58^. Therefore, in this study, the dual-drug delivery system (pGO-Pt/DOX) was PEG-functionalized and the size was suitable for the EPR effect. The EPR effect was then indicated by the *in vitro* cell uptake assay (Fig. 4E), and this in turn gave a comparable IC_50_ value between the pGO-Pt/DOX group and the 3-fold higher amount of the Pt/DOX group (Table 2). These results revealed that the dual-drug delivery system could satisfy the harsh request of the long circulation time in the plasma, maintain their antitumor effect.

### The superiority of the dual drugs for the cancer cell

In recent years, combination chemotherapy has been adopted as a standard clinical strategy against many types of cancer^14^. In this study, Pt and DOX were chosen to demonstrate the possibility of combination chemotherapy simultaneously on graphene. The carboxylic acid group at the edge of GO allowed amide interactions with Pt, and the basal plane of GO, which was mainly composed of polyaromatic networks, allowed π-π stacking of DOX (Fig. 1). These two drugs are widely used in clinic for combination chemotherapy with a synergistic effect^11,59^. Figure 4A–D demonstrated that the anticancer activity could be improved by using the simultaneous delivery ability. Figure 5 showed that this dual-drug delivery system induced not only apoptosis but also necrosis of cancer cells. Furthermore, *in vivo* Ki67 staining (Fig. 7) indicated that the cancer cell proliferation has been inhibited by the use of a dual-drug delivery system. The mechanisms might be explained as follows (Fig. 1B): First, the anticancer effects of Pt could be to generate DNA lesions, then inducing cell senescence or apoptosis. Pt was also shown to trigger cell necrosis by arousing cytotoxic effects from both nuclear and cytoplasmic signaling pathway^60^. On the other hand, the anticancer effect of DOX could be to prevent DNA replication, inhibiting cell proliferation activity. DOX was also shown to damage mitochondrial DNA (mtDNA) and led to lower tumor energy supply^38^. Therefore, although the molecular mechanisms underlying the cytotoxic potential of Pt and DOX remain poorly understood, pGO-Pt/DOX dual-drug delivery system has presented a more excellent anticancer effect than either single drug or free drugs.

## Materials and Methods

### Materials

All chemical reagents used for this study were of analytical grade or above. Graphene oxide (GO) was purchased from Nanjing XFNANO (Nanjing, China). 4-arm PEG-amine (aver. Mw2000 Da) was obtained from J&K Scientific Ltd (Beijing China). Cis-Diammineplatinum(II) dichloride (cisplatin), N-(3-Dimethylaminopropyl)-N′-ethylcarbodiimide hydrochloride (EDC) and N-hydroxysuccinimide (NHS) were purchased from Sigma Aldrich (Saint Louis, USA). Doxorubicin hydrochloride (DOX) was purchased from Beijing Mesochem Technology Co., Ltd. (Beijing, China). Other reagents were obtained from Chengdu Jinniu Chemical Reagent Co. (Chengdu, China) and used as received without further purification. All solutions were prepared with ultra-pure water (18.2 MU/cm) from a Millipore system.

The murine fibroblasts cell line (L929), the human squamous cell carcinoma cell line (CAL-27) and human breast cancer cell line (MCF-7) were supplied by State Key Laboratory of Oral Diseases (Chengdu, China). L929 and CAL-27 cells were grown in Dulbecco’s modified Eagle’s medium (DMEM, Gibco, USA) and MCF-7 cells were grown in RPMI-1640 medium (Gibco, USA), and both medium were supplemented with 10% FBS (HyClone, USA), 100 U/mL penicillin, 100 μg/mL streptomycin (Sigma, USA), and cultured at 37 °C under a humidified atmosphere containing 5% CO2, respectively. 2-(4-Amidinophenyl)-6-indolecarbamidine dihydrochloride (DAPI) was purchased from Beyotime (Shanghai, China). Cell counting kit-8 (CCK-8) was purchased from Dojindo Molecular Technologies, Inc. (Rockville, USA). Annexin V-FITC/PI Apoptosis Detection Kit was purchased from KeyGEN Biotech Co., Ltd. (Nanjing, China). Hematoxylin and eosin (H&E) stains were purchased from BBC Biochemical. Anti-Ki67 antibody was obtained from Abcam (Cambridge, USA).

Six-week-old female athymic nude mice were obtained from the Laboratory Animal Centre of Sichuan University (Chengdu, China), and were housed in a temperature-controlled environment with 12 h light/dark cycles. All animal procedures were approved by the Institutional Animal Care and Ethics Committee of Sichuan University (Chengdu, China), in accordance with the ARRIVE guidelines. Efforts were taken to ensure that the guiding principles of the three R’s were followed.

### Characterizations

Transmission electron microscopic (TEM) images were performed using a FEI Tecnai G2 F20 S-TWIN high-resolution transmission electron microscope operating at 200 kV. Atomic force microscopy (AFM, SPM-9600, Shi-madzu, Japan) images were obtained on a Si substrate. The Zeta potential were determined by a Malvern Zeta Sizer (Malvern, NanoZS, UK). Fourier transform infrared (FTIR) spectra were recorded on a Nicolet 6700 FTIR spectrometer (Thermo Fisher Scientific, USA). Raman spectra were taken with a LabRAM HR-800 micro spectrometer system equipped with a 514.5 nm Arþ laser. UV-Vis spectra were performed by a UV-Vis spectrometer (Lambda 35, PerkineElmer, USA). The platinum contents were measured by inductively coupled plasma-optical emission spectroscopy (ICP-OES, Arcos, Germany).

### Synthesis and characterization of PEGylated nano-graphene oxide (pGO)

PEGylated nano-graphene oxide (pGO) was first synthesized following a previously published protocol^21,61^. Briefly, nano-sized graphene oxide (NGO) were firstly obtained by soaking 10 mg GO into 5 mL deionized water and sonicating at the power of 570 W for 30 min, followed by adding the sodium hydroxide (NaOH) solution (1.2 g) and chloroacetic acid (CH_2_ClCOOH)(1.0 g). The NGO was then collected, re-dispersed in deionized water and sonicated again at 570 W for 2 h to give a clear solution in an ice bath^61^.

The NGO dispersion (1 mg/mL, 10 mL) was mixed with 10 mg of PEG in a flask by sonicator for 30 min. Then 4 mg of EDC and 6 mg of NHS were added and the mixture was stirred overnight. The PEGylated nano-graphene oxide (pGO) was filtered with MWCO of 100 kDa Millipore filters (Millipore, Bedford, MA, USA) and dried under vacuum. Eventually, the obtained pGO solution was acquired and monitored by zeta-potential analysis, TEM image, AFM image and UV-Vis spectra (Fig. SI).

### Synthesis and characterization of pGO-Pt

As described in Fig. 1, cisplatin (Written as Pt (II)) was firstly oxidized with H_2_O_2_ then reacted with succinic anhydride to form c,c,t-Pt(NH_3_)_2_Cl_2_(OOCCH_2_CH_2_COOH)(OH) (abbreviated as Pt(IV) or Pt) followed the protocol reported from previous study^19^. To synthesize the pGO-Pt nanoparticles, EDC and NHS were firstly added into 0.1 mg/mL pGO (m(EDC):m(NHS):m(pGO) = 0.4:0.6:1), followed by the addition of Pt, then the mixture was bath sonicated for 30 min and stirred vigorously overnight at room temperature. The as-prepared pGO-Pt was purified by dialysis (MW cutoff of 3500) for 48 h and repeated ultrafiltration, and dried under vacuum. All the supernatant and the washing liquor after dialysis and ultrafiltration were collected. Afterwards, a series of evaluations (Zeta-potential, AFM, TEM, Raman spectrometry, UV-Vis spectra, and FTIR spectroscopy) were conducted to characterize the resultant pGO-Pt nanoparticles. All experiments based on Pt were protected from light to avoid its decomposition.

### Synthesis and characterization of pGO-Pt/DOX

To synthesize the dual-drug delivery system, DOX (10 mg) was dissolved into 10 mL deionized water solution. Then the solution of DOX was added to the solution of pGO-Pt, and the pH value was adjusted to 8 with NaOH solution^61^. The mixture was stirred at room temperature under light-sealed conditions for 24 h. After the reaction was completed, the products were purified by dialysis for 48 h and repeated ultrafiltration, then dried under vacuum. All the supernatant and the washing liquor after dialysis and ultrafiltration were collected. Finally, the pGO-Pt/DOX dual-drug delivery system was evaluated by zeta-potential analysis, AFM, TEM, and UV-Vis spectra. All experiments were protected from light to avoid drugs decomposition.

### Drug loading efficacy (DLE) of Pt and DOX

To determine the DLE, each series of experiments was carried out in triplicate under the corresponding test conditions (Supporting Information, SI). According to the DLE curve, we optimized the initial amount of DOX to make the finally weight ratio of DOX: Pt: pGO = 0.376: 0.376: 1 (Fig. 3C).

### *In vitro* dual-drug release from the pGO-Pt/DOX nanoparticles

The pGO-Pt/DOX nanoparticles (6 mg) were dispersed in 6 mL of phosphate buffer solution (PBS, pH = 7.4) and the dispersion was divided into two equal aliquots. The pGO-Pt/DOX samples used for release study were transferred into a dialysis bag, which were kept in 200 mL of aqueous solution under constant stirring with pH 7.4 and 5.3, respectively. At certain time intervals, a portion of release medium (2 mL) was taken out for characterization and then fresh buffer (2 mL) was added to keep the volume constant. The amount of released Pt was determined by ICP-OES and the amount of released DOX was measured by UV–vis spectroscopy at 480 nm.

### Cell viability and cytotoxicity *in vitro*

#### Cell viability of pGO

To investigate cell viability of the drug carrier material (pGO), the murine fibroblasts cell line (L929) was seeded into 96-well plate with a concentration of 1 × 10^4^ cells/well and incubated for 24 h. After replacing the medium, different concentration of pGO was added into the plates at concentrations ranged from 0 to 100 μg/mL. The cells were incubated for 24, 48, and 72 h. Then, cell viability was assessed by cell counting kit-8 assay (CCK-8) according to the protocol suggested by the manufacturer. The optical density (OD) of the samples at a wavelength of 450 nm was measured by microplate reader (Thermo, Varioskan Flash). A triplicate analysis was induced from three independent experiments (n = 3).

#### Cytotoxicity of pGO-Pt/DOX nanoparticles

To assess the cytotoxicity and tumor targeting of pGO-Pt/DOX nanoparticles, CAL-27 and MCF-7 cell lines were chosen to incubate with PBS, pGO, pGO-Pt, pGO-DOX, pGO-Pt/DOX, and free drugs (Pt/DOX mixture, m(Pt):m(DOX) = 1:1) *in vitro*. In generally, CAL-27 and MCF-7 cells were firstly seeded and incubated at 37 °C for 24 h. Then PBS, pGO, pGO-Pt, pGO-DOX, pGO-Pt/DOX, and Pt/DOX were administrated at five final concentrations of 1, 2, 5, 10, and 20 μg/mL. After incubated for 24 and 48 h, the cell cytotoxicity was determined by CCK-8 assay according to the manufacturer suggested procedures. A triplicate analysis was induced from three independent experiments (n = 3). After the determination of half maximal inhibitory concentration (IC_50_), we chose CAL-27 cell line in the following study.

#### *In vitro* cellular uptake

To investigate the cellular uptake of the dual-drug delivery system by means of fluorescence microscopy, CAL-27 cells were seeded into 24-well plates at 1 × 10^5^ cell/mL. Twenty-four hours later, each well was incubated with 5 μg/mL DOX-labeled pGO-Pt/DOX and Pt/DOX mixture for 24 h at 37 °C. At the end of the incubation period, the solutions were removed, and the cells were stained with DAPI and rinsed three times with cold PBS. Then, the cells were observed under a fluorescence microscope (Leica, Wetzlar, Germany).

#### Cell apoptosis assay

The quantitative analysis of cell apoptosis induced by dual-drug delivery system was performed by Annexin V-FITC/PI double staining. CAL-27 cells were seeded in 6-well plates at a density of 5 × 10^5^ cell/mL. After 24 h incubation, cells were treated for 4 h with PBS, pGO, pGO-Pt, pGO-DOX, pGO-Pt/DOX, and Pt/DOX mixture, the final concentration of the drugs or PBS was 5 μg/mL. Then, treated cells were harvested, washed with cold PBS, suspended in 400 μL of binding buffer, and stained with 5 μL of Annexin V-FITC for 15 min and 5 μL of PI for 5 min at 4 °C in the dark. The stained cells were then analyzed by a flow cytometer (Beckman Coulter, Fullerton, CA, USA).

### *In vivo* antitumor efficacy

#### Animal preparation

All animal procedures were approved by the Institutional Animal Care and Ethics Committee of Sichuan University (Chengdu, China), in accordance with the ARRIVE guidelines. The CAL-27 tumor models were generated by the subcutaneous injection of 1 × 10^6^ cells into the dorsal right side of six-week-old female nude mice. When the tumor volume reached 50–100 mm^3 ^^62,63^, the mice were divided into four groups (five mice in each group), minimizing the differences in weights and tumor sizes in each group. The mice were subjected to intravenous administration with (a) PBS (200 μL), (b) pGO (10 mg/kg, 200 μL), (c) pGO-Pt/DOX (10 mg/kg, 200 μL) and (d) Pt/DOX mixture (m(Pt):m(DOX) = 1:1, 10 mg/kg, 200 μL) every 3 days, for a total of seven injections. Tumors were measured and mice were weighed every two days over a period of 3 weeks. The volume of the tumor (V) was calculated using the following formula^64^:

$$V(m{m}^{3})=(L{W}^{2})/2$$

where V is the tumor volume, L is the tumor length, and W is the tumor width. In addition, the drug accumulation of pGO-Pt/DOX and Pt/DOX mixture were performed by a quantification analysis of Pt and DOX amounts in tumor tissue 12 h post-injection.

#### Analysis of tumor and blood chemistry

Three weeks later, the mice were anesthetized and blood samples were collected from the vein of the fundus oculi before being euthanatized. Liver function was determined by the alanine aminotransferase (ALT), aspartate aminotransferase (AST), and alkaline phosphatase (ALP); nephrotoxicity was evaluated with blood urea nitrogen (BUN); cardiac damage was assayed by lactate dehydrogenase (LDH) and creatinine (CK) using a Biochemical Autoanalyzer (Type AU680, Beckman Coulter, USA)^65,66^.

Major organs including tumor, heart, liver, spleen, lung, and kidney were harvested and fixed in 4% paraformaldehyde overnight at 4 °C. These samples were then dehydrated through an ascending ethanol series prior to paraffin embedding. Eight micrometer sections were cut and collected on Superfrost-plus slides. Tissue sections prepared for histology and immunohistochemistry were performed by one individual and then were quantified by a blinded individual. Hematoxylin and eosin (H&E) staining for the major organs and immunohistochemistry for tumors were performed as previously described^67^, and a rabbit anti-mouse monoclonal antibody Ki67 was used as the primary antibody. To quantify Ki-67 expression, areas in each tumor sample were randomly selected and the Ki-67 labeling index was calculated as the number of Ki-67-positive cells/total number of cells. All images were acquired by using a Nikon microscope (Eclipse 80i, Japan).

## Conclusion

In this study, we developed a dual-drug delivery system using PEG-functionalized graphene oxide (pGO) and antitumor drugs (Pt and DOX). We employed zeta-potential, TEM, and Raman, UV-Vis, and FTIR investigations to characterize the as-prepared pGO-Pt/DOX, and the results exhibited that nano-sized pGO-Pt/DOX was successfully fabricated. The drug delivery efficacy of Pt was enhanced through the introduction of pGO, and the final weight ratio of DOX: Pt: pGO was optimized to 0.376: 0.376: 1. Drug release results indicated that both Pt and DOX release kinetics from pGO-Pt/DOX nanoparticles were pH-dependent. *In vitro* studies suggested that pGO-Pt/DOX nanoparticles were effectively delivered into tumor cells, indicated the most prominent cell apoptosis and necrosis, and then exhibited a higher growth inhibition property than the single drug delivery system or free drugs. *In vivo* data confirmed that pGO-Pt/DOX dual-drug delivery system attenuated the toxicity of Pt and DOX to normal organ compared to free drugs. The tumor inhibition data, histopathology observations, and immunohistochemical staining have confirmed that the dual-drug delivery system presented more excellent anticancer effect than free drugs. This study demonstrated that the combination of Pt and DOX onto PEG-functionalized nano-sized GO afforded numerous advantages for tumor therapy such as minimizing systemic toxicity, controlling drug release under acid environment of tumor, and enhancing therapeutic efficacy, implying this dual-drug delivery system has great potential for clinical applications.

## Supplementary information

supporting information.
