# Supplementary material for: PEGylated nano-graphene oxide as a nanocarrier for delivering mixed anticancer drugs to improve anticancer activity
Source: Sci Rep. 2020 Feb 17;10:2717. doi: 10.1038/s41598-020-59624-w (PMC7026168; doi:10.1038/s41598-020-59624-w)
Supplement: Supplementary file 1 — supporting information. [file 41598_2020_59624_MOESM1_ESM.docx]

**Supporting information**

**For**

PEGylated nano-graphene oxide as a nanocarrier for delivering mixed anticancer drugs to improve anticancer activity

Xibo Pei^1 a *^, Zhou Zhu^1 a^, Zhoujie Gan^1^, Junyu Chen^1^, Xin Zhang^1^, Xinting Cheng^1^, Qianbing Wan^1^, Jian Wang^1 *^

^1.^ State Key Laboratory of Oral Diseases, National Clinical Research Center for Oral Diseases, Department of Prosthodontics, West China Hospital of Stomatology, Sichuan University, Chengdu, Sichuan 610041, China

*** Corresponding author:**

Xibo Pei; e-mail: xbpei@hotmail.com Tel:+86-13666287060 Fax: +86-28-85501450

Jian Wang; e-mail: ferowang@hotmail.com Tel:+86-13541202602 Fax: +86-28-85501450

Xibo Pei and Jian Wang, Sichuan University, No. 14, Section 3, South Peoples Road, Chengdu 610041, China.

^a^ These authors contributed equally to this work.

**KEYWORDS:**

Graphene oxide; Drug delivery; Cisplatin; Doxorubicin; Combination chemotherapy

**Materials and Methods**

2. Materials and methods

2.1. Drug loading efficacy (DLE) of Pt and DOX

To determine the DLE, each series of experiments was carried out in triplicate under the corresponding test conditions.

2.1.1 The DLE of Pt

Firstly, the DLE of pGO for Pt was tested by changing the initial concentration ratio between Pt and pGO (Table SI, from 1:1 to 9:1). The residual Pt was evaluated by ICP-OES using the supernatant and the washing liquor collected after dialysis and ultrafiltration. The DLE was calculated form the following equation:

DLE (mg/mg)=[ W_Pt_- W_Pt’_]/W_pGO_

where W_Pt_ is the initial amount of Pt, W_Pt’_ is the residual amount Pt that is not attached onto the pGO, W_pGO_ is the initial amount of pGO.

The saturated DLE of Pt was 0.376 mg/mg (Fig. 3C), and this data were chosen for the subsequent experiments.

Table SI The initial concentration ratio between Pt and pGO.

| Groups | 1 | 2 | 3 | 4 | 5 | 6 | 7 | 8 | 9 |
| --- | --- | --- | --- | --- | --- | --- | --- | --- | --- |
| Pt(mg) | 0.1 | 0.2 | 0.3 | 0.4 | 0.5 | 0.6 | 0.7 | 0.8 | 0.9 |
| pGO(mg) | 0.1 mg for each group | | | | | | | | |
| Pt:pGO | 1:1 | 2:1 | 3:1 | 4:1 | 5:1 | 6:1 | 7:1 | 8:1 | 9:1 |

2.1.2 The DLE of DOX

Then, the DLE of DOX was studied by changing the initial concentration ratio between DOX and pGO (Table SII, from 1:10 to 9:10). The residual DOX was calculated by using the the calibration curves of DOX standard solutions obtained by UV–Vis spectroscopy at a wavelength of 480 nm.

DLE (mg/mg)=[ W_DOX_- W_DOX’_]/W_pGO_

where W_DOX_ is the initial amount of DOX, W_DOX’_ is the residual amount of DOX that is not attached onto the pGO-Pt, W_pGO_ is the initial amount of pGO in pGO-Pt nanoparticles.

According to the DLE curve, we optimized the initial amount of DOX to make the finally weight ratio of DOX: Pt: pGO = 0.376: 0.376: 1 (Fig. 3C).

Table SII The initial concentration ratio between DOX and pGO.

| Groups | 1 | 2 | 3 | 4 | 5 | 6 | 7 | 8 | 9 |
| --- | --- | --- | --- | --- | --- | --- | --- | --- | --- |
| DOX(mg) | 0.1 | 0.2 | 0.3 | 0.4 | 0.5 | 0.6 | 0.7 | 0.8 | 0.9 |
| pGO | 1 mg for each group | | | | | | | | |
| Pt | The DLE of Pt was 0.376 mg/mg | | | | | | | | |
| Pt:pGO | 1:10 | 2:10 | 3:10 | 4:10 | 5:10 | 6:10 | 7:10 | 8:10 | 9:10 |

**Results - Supporting figures**


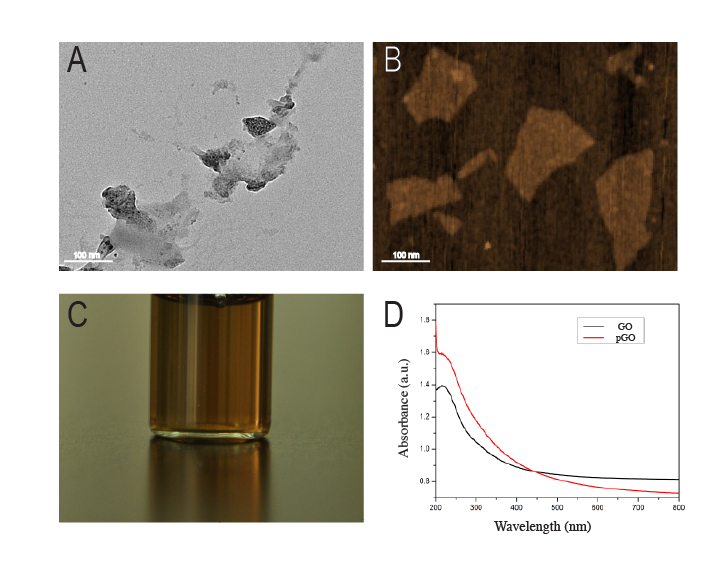


Fig. S1 Nano-sized pGO were successful fabricated. (A) TEM image of nano-sized pGO. (B) AFM image of nano-sized pGO. (C) Solution of pGO. (D) UV-vis spectra of GO and pGO.


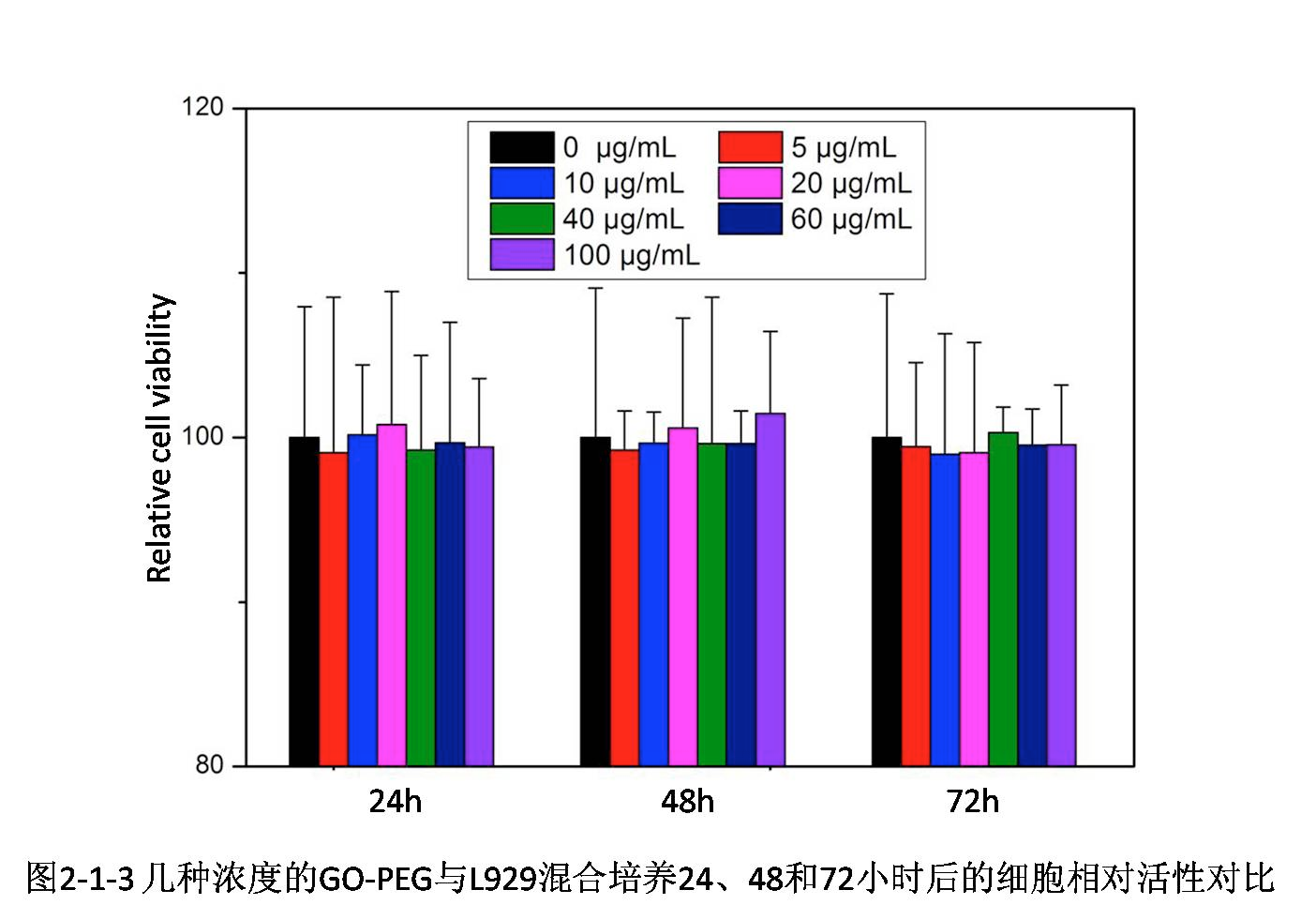


### Fig. S2 Cell viability of pGO. To investigate cell viability of the drug carrier material (pGO), the murine fibroblasts cell line (L929) was seeded into 96-well. Different concentration of pGO was added into the plates at concentrations ranged from 0 to 100 μg/mL. The cells were incubated for 24, 48, and 72 h. CCK-8 results showed that pGO revealed a high viability to L929 cells at 0 - 100 μg/mL after 24 and 48 h, and even after 3 days of incubation, the L929 cell viability still reached 94.3 % at a concentration as high as 100 μg/mL.
